# Thermal expansion coefficients in Invar processed by selective laser melting

**DOI:** 10.1007/s10853-017-1169-4

**Published:** 2017-05-11

**Authors:** Neil J. Harrison, Iain Todd, Kamran Mumtaz

**Affiliations:** 1 Department of Mechanical Engineering, University of Sheffield, Sheffield, UK; 2 Department of Materials Science and Engineering, University of Sheffield, Sheffield, UK

## Abstract

This work investigates whether the unique low thermal expansion property of Invar (64Fe–36Ni) is retained after processing using the additive manufacturing process selective laser melting (SLM). Using this process, near-full-density components (99.96%) were formed by melting thin (20 μm) layers of powdered Invar (15–45 μm particle size). The mechanical properties of SLM Invar were comparable to that of cold-drawn Invar36^®^; however, the thermal coefficient of expansion was observed to be a lower value and negative up until 100 °C. This negative value was attributed to residual stress in the as-deposited parts. The low thermal expansion property of Invar was still maintained when processed using a non-conventional layer-based additive manufacturing technique.

## Introduction

Invar and its variants are Fe–Ni alloys (based around a 64Fe–36Ni composition) which display very low coefficients of thermal expansion (CTE) for temperatures up to around 200 °C. The phenomenon, known as the Invar effect, depends on the energetic state of the nearest neighbour Fe–Fe bonds. Rancourt [1] found that up to one in five magnetic exchange bonds in Invar were energetically unsatisfied, and as such, it was a heavily frustrated system. Calculations of the ground state magnetovolume properties revealed that the unsatisfied bonds display the opposite magnetovolume action to satisfied bonds. The consequence of which is a negative magnetovolume force opposing the thermal expansion of the alloy. The effect is strong up to 100 °C but begins to weaken with increasing temperature as atomic vibration increases. At the Curie point (279 °C for Invar [2]), the material becomes completely paramagnetic and normal thermal expansion resumes.

Because of its uniquely low thermal expansion properties, Invar is ideal for applications where high-dimensional stability is required over atmospheric temperature ranges. It is most commonly used in high-precision instrumentation such as altimeters and time-keeping devices. It possesses tensile properties similar to low-grade steels, making it more appropriate than polymers for any application where loading is required.

Selective laser melting (SLM) is an additive manufacturing (AM) process in which layers of metallic powder are selectively melted and fused by a high-powered laser to form near-fully dense 3D components. The method of layered fabrication, combined with the high precision of laser melting, allows for a greatly expanded design freedom with minimal feedstock waste. It is increasingly being used in high value markets for the production of various aerospace, automotive and medical components.

This work builds on the work by Qiu et al. [3] who investigated the microstructure and properties of selective laser-melted Invar36^®^. Qiu reported that the as-processed microstructure comprised of columnar vertically orientated γ grains, interspersed by nano-precipitate α-phase. The as-processed material displayed anisotropic tensile behaviour, with specimens built in the horizontal orientation displaying superior yield strength and UTS to those built in the vertical; but elongation was superior in the vertical. This behaviour has been noted in other investigations for nickel-base alloys [4–6] processed by metal powder bed fusion. In addition, it was reported that SLM-fabricated Invar36^®^ displayed the same low thermal expansion properties up to 300 °C as conventionally manufactured Invar36^®^. It was reported that there was a marginal reduction in thermal expansion after heat treatment when tested in the vertical orientation.

In this work, more focus is placed on the effect of the SLM process on the thermal expansion of Invar and similar alloys, and what influence the process characteristics, if any, have on the magnetovolume phenomenon. Analysis of microstructure and mechanical properties is also conducted for reference.

## Experimental methodology

SLM processing parameters, such as laser energy density and beam velocity, were optimised for the fabrication of fully dense parts with minimal defects, and the physical properties of the resultant fabricated Invar components were investigated. Samples were fabricated on a Renishaw SLM125 using a modulated 200 W ytterbium fibre laser with a 125 × 125 × 125 mm build volume. The Invar powder feedstock (sourced from LPW Technology, UK) was manufactured using gas atomisation to attain a consistent spherical morphology and was sized to 15–45 μm with a composition of 63.7 wt% Fe, 35.8 wt% Ni, 0.47 wt% Mn and 0.032 wt% C.

In previous optimisation trials for Fe–Ni based alloys, it was established that a layer thickness of 20 μm and hatch spacing of 0.09 mm were optimal for achieving maximum density [7] and thus would be set as fixed parameters for the remainder of this investigation. The scan strategy used was a raster pattern with a 67° rotation after each layer, known as a ‘meander’ strategy; it is designed to maximise interlayer density and reduce thermal stress build-up within the parts. Parameter optimisation in this study focussed on laser power (LP), point distance (PD) and exposure time (ET). The laser scan motion on the Renishaw SLM125 is a ‘point-to-point’ traverse, where the laser path is comprised of a series of single exposure points (of time ET) separated by a point distance (PD), as opposed to a continuous laser exposure which moves at a ‘beam velocity’ along the full length of the scan path. PD and ET can be combined, with the addition of idle time, to give an apparent scan velocity. Using Design of Experiment (DOE) software Minitab^®^, an experimental plan was devised to create samples from a range of SLM parameters consisting of minimum and maximum values of 180–200 W, 90–150 μs and 50–90 μm for LP, ET and PD, respectively. The central point of 190 W, 120 μs and 70 μm was repeated three times for validation purposes. The density results from the initial experiment were input into Minitab^®^, and a second full-density solution parameter set was calculated.

**Figure 1:**
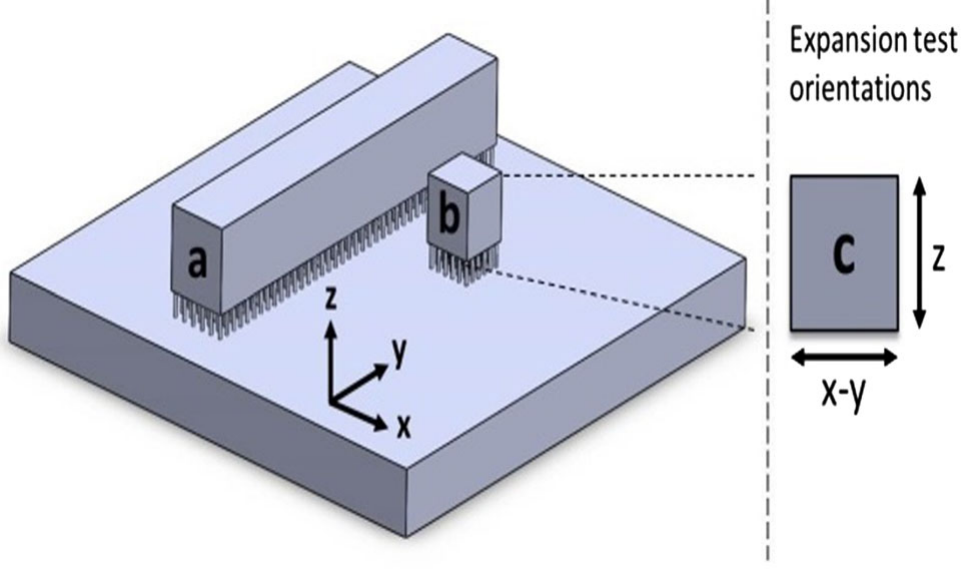
Schematic demonstrating build orientation of tensile component cuboids (**a**) and thermal expansion cubes (**b**), as well as test orientations for thermal expansion analysis (**c**)

Percentage density was optically calculated using micrographs of sectioned samples by taking area fraction measurements of the binary images on Image J software. Samples were mounted in conducting resin, ground and polished using SiC pads in grades from P800-P2500 and diamond suspension polish from 3 to 1 μm, to reveal polished vertical and horizontal sections. Two sections were prepared of each sample, with three micrographs taken for each section—allowing for calculation of mean values with standard error. This method is preferred over displacement techniques as it reveals the quantity and morphology of porosity that may exist within a sample. Samples for microstructural analysis were etched using a 2% Nital solution (98 ml of IMS with 2 ml of nitric acid). Tensile rounds were machined from cuboids fabricated in the x–y orientation, see Fig. 1, to ASTM A370 standard sizing. Tensile components built in x–y (built with the gauge length in the x–y plane, rather than z plane) have been found to display slightly higher tensile strength than those built in z [8]. Therefore, the x–y orientation represents a maximum tensile strength for the as-deposited state. It is noted that the same investigation [8] found that elongation was greater in tensile components built in the z plane; therefore, x–y plane components represent a minimum for elongation. Ultrasonic analysis was conducted on as-deposited samples to determine Young’s modulus, independently of the tensile results, and was performed on an Olympus EPOCH 600 Ultrasonic Flaw Detector. Young’s modulus is calculated from a measured longitudinal wave sound velocity and known material density (8.105 g/cm^3^ as measured by helium gas pycnometry on a Micromeritics AccuPyc 1340 Pycnometer) and Poisson’s ratio (0.3) [2]. The samples were fabricated as 8 mm cubes, with the parallel surfaces ground to ensure sufficient contact, and measurements were taken four times per sample for two samples. Thermal expansion analysis was conducted on a Perkin-Elmer Diamond TMA; the CTE measurements were taken on the same 8 mm cubes from the ultrasonic measurements. Testing was conducted for both x–y and z orientations, see Fig. 1, as per ASTM standard E831 using a heating rate of 5 K per min, with three cycles per sample. The sensitivity of the device is 0.02 μm.

## Results and analysis

Power, exposure time and point distance are all controlling variables of laser energy, either per unit time or unit length. Plotting density against one of the three is nonsensical as it neglects the influence of the other two. As such, LP, ET and PD have been dimensionally reduced into a single parameter of 1D line energy density. This is an appropriate use of energy density since layer thickness and hatch spacing are fixed; thus, any variation in density is purely a consequence of the absorbed energy. If the energy density is considered over a set length, it can be described as ${\text{Energy per point}} \times {\text{Points per unit length}}$ or $P \times {\text{ET}} \times 1/{\text{PD}}$, and this becomes:

1 $$ \frac{{Pt_{\exp } }}{{x_{\text{PD}} }} \equiv \frac{Q}{l} $$

where *P* is the power, *t*_exp_ is the exposure time, *x*_*PD*_ is the point distance, *Q* is the energy, and *l* is the unit length. Note this relationship was first used in a previous publication by Harrison et al. [7].

**Figure 2:**
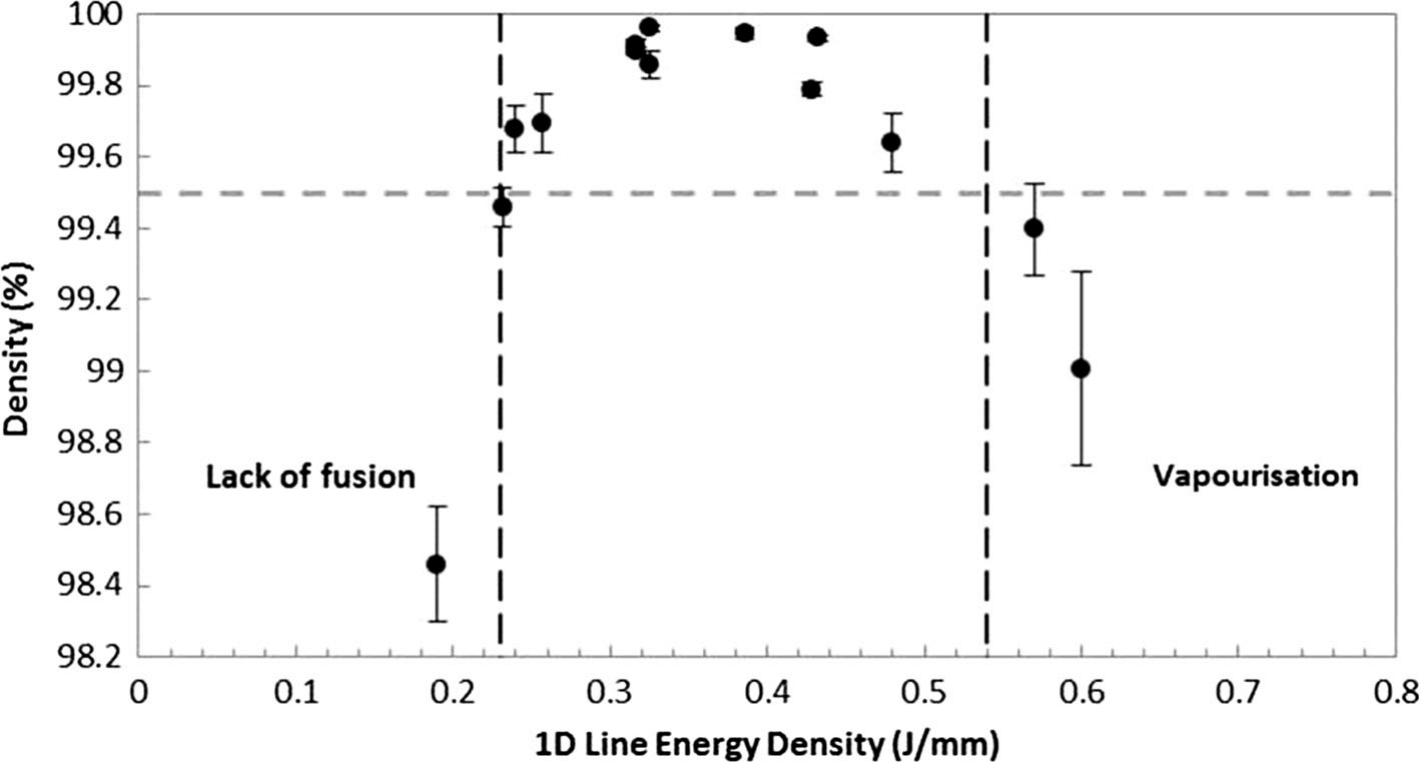
Percent density against 1D line energy density. *Grey dashed line* set at 99.5%, greater values were considered fully dense

Figure 2 indicates a strong relationship between part density and 1D line energy density, with a global maximum located between 0.35 and 0.40 J/mm. The highest observed density was 99.96% with a 1D energy density of 0.33 J/mm, of which the scan parameters were LP 190 W, ET 120 μs and PD 70 μm. There were 10 samples which achieved densities ≥99.5%.

**Figure 3:**
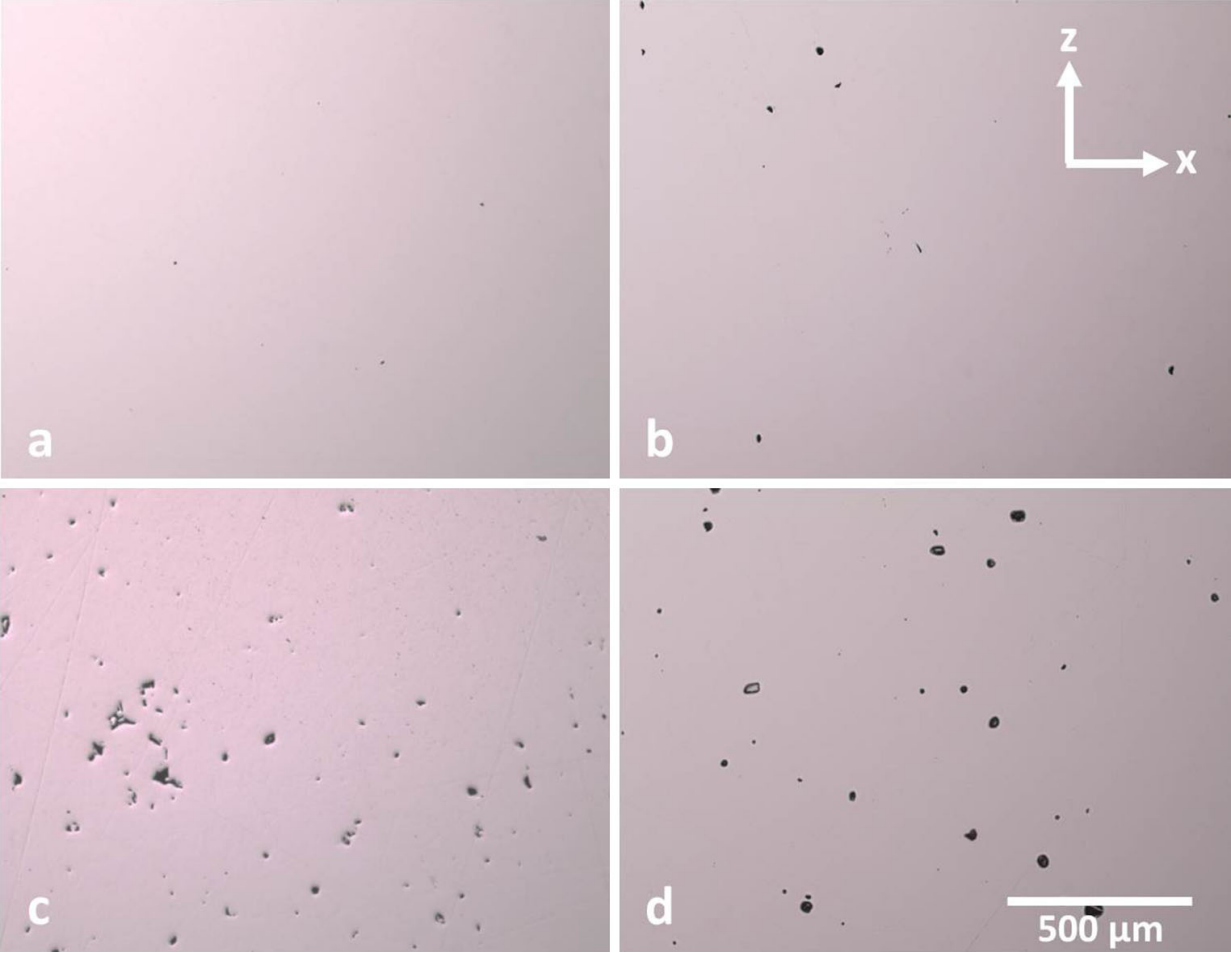
**a** 99.96% dense, **b** >99.5% dense, **c** example of lack of fusion porosity in <99.5% dense sample, **d** example of vapourisation porosity in <99.5% dense sample. *Scale bar* and axis apply to all images

Energy densities which fail to produce samples with density of ≥99.5% have been split into two regions according to the type of porosity that was observed (shown in Figs. 2, 3). Lack of fusion occurs when the energy density is not sufficient to generate full melting of the powdered layer, leading to pockets of unmelted particles and, in extreme cases, delamination between previously processed layers. In cases of high energy densities, the generated surface temperature can exceed the evaporation temperature of the alloy, leading to vapourisation recoil which ejects particles and molten material from the heat-affected zone resulting in large irregular voids. Gas may also be trapped within these voids leading to large spherical pores.

**Figure 4:**
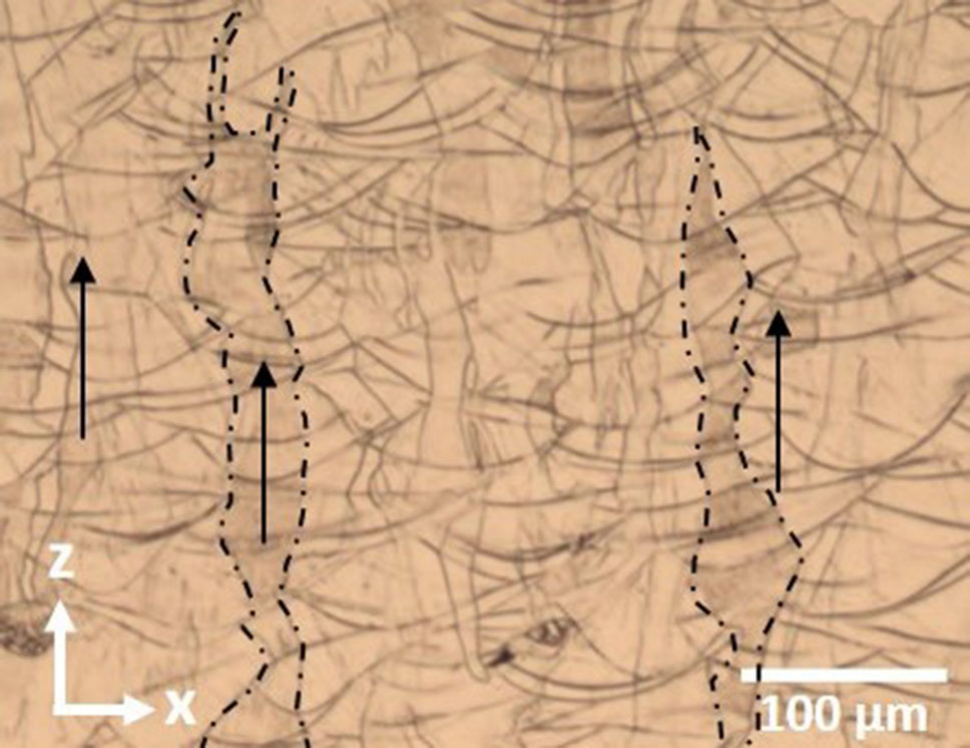
Optical micrograph of etched sample—note highly directional columnar grains highlighted by* dashed lines*. The growth direction has also been highlighted by *black arrows*

In Fig. 4, it is observed that the grains have grown epitaxially and transcend the layers, with vertical grain length being in the order of 200–300 μm. The grain structure is columnar and orientated in the build z-direction and is in agreement with that reported in other investigations [9–13], including Qiu et al. [3]. The meander scan strategy used in this investigation has resulted in more grain intersections than if processed using a raster pattern without rotation, which would have allowed for more continual epitaxial growth. Chen et al. [14] describe how crystallographic orientation selection is influenced by the scan direction-dependent heat flux and that the epitaxial growth is dependent on the angle between the grain growth and (scan direction-dependent) heat flux with high angles likely to result in stoppage due to the grain impinging on another and low angles more readily continuing to the track surface. The meander strategy likely increases the chance high angles being formed due to the rotation of scan direction after each layer. The result is a less ordered grain structure, made up of large columnar grains interspersed with small more irregular grains. This structure was also observed in an investigation by Harrison et al. [7] where the same meander scan strategy was used on a nickel-base superalloy. The dark arcs observed in Fig. 4 are the melt traces, or base of the individual melt pools, which have been highlighted by the etching process indicating minor solute segregation.

Table 1 displays the results of the tensile testing of SLM Invar samples in comparison with the literature values for cold-rolled and annealed Invar36^®^. Both UTS and offset yield strength of the SLM-fabricated Invar exceed that of the annealed, which is consistent with other findings for solid solution alloys [7, 15, 16] and is more comparable to cold-drawn Invar36^®^. The mechanical properties are also consistent with those reported by Qiu et al. [3] for the same conditions. Elongation is marginally reduced for SLM Invar over annealed; however, the reduction of area is greater, implying fewer internal defects.

**Table 1:** Tensile property comparison between SLM-fabricated Invar and cold-drawn and annealed Invar36^®^[2]

	UTS (MPa)	*σ*_0.2_ (MPa)	E (GPa)	El (%)	R of A (%)
SLM’d *x–y* axis test direction	516.7 ± 1.4	440.7 ± 0.2	137.5 ± 4.2^a^	30 ± 0.5	73.7 ± 1.7
Cold-drawn Invar36^®^	621	483	137–145^b^ [17]	20	60
Annealed bar Invar36^®^	448	276	137–145^b^ [17]	35	65

^a^Results from both tensile and ultrasonic testing

^b^Values taken from CES Edupack database [17] as omitted from Carpenter Technology Corporation datasheet [2]

**Figure 5:**
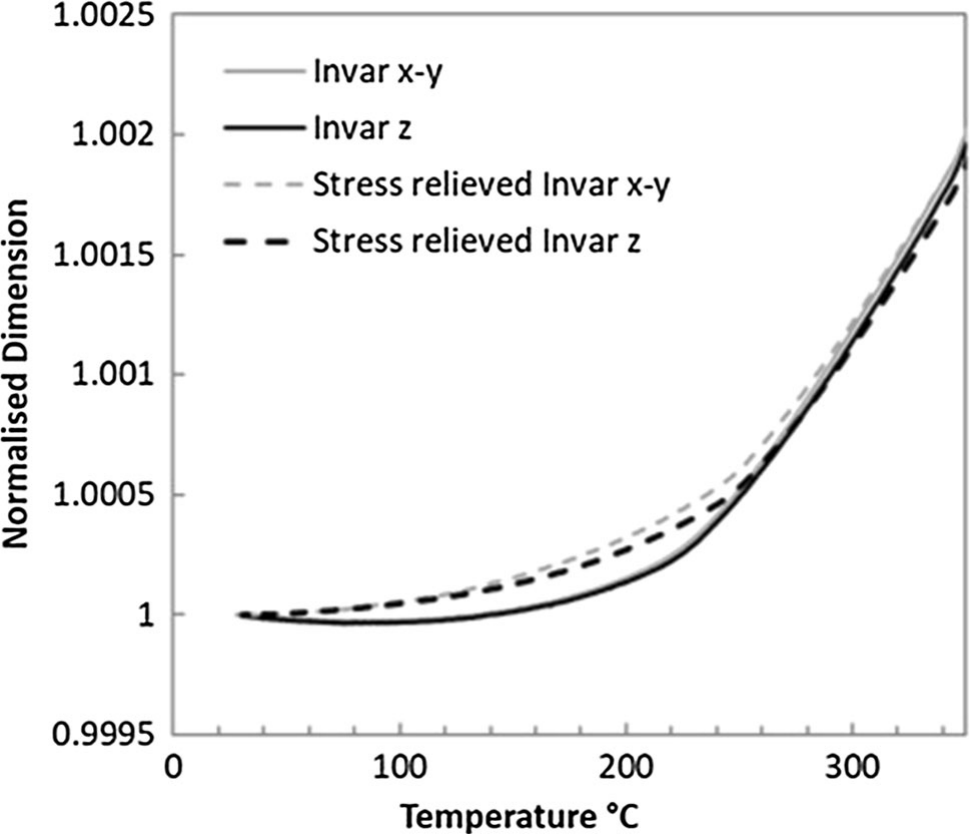
Normalised thermal expansion for x-y and z test orientations of SLM Invar both stress-relieved and in as-deposited state

Figure 5 displays the linear expansion curve of SLM Invar for a temperature range of 30–300 °C, as well as those for stress-relieved Invar samples which will be discussed in "Observed reduction of thermal expansion coefficient" section. The values have been normalised against the room temperature dimension of each sample for fair comparison. Table 2 gives the mean coefficient of thermal expansion *α*_CTE_ for five temperature ranges, as well as comparison values for annealed Invar36^®^.

**Table 2:** Mean coefficient of thermal expansion *α*_CTE_ for SLM-fabricated Invar, Invar 36^®^[2], and stress-relieved SLM-fabricated Invar

Temp (°C)	30–100	30–150	30−200	30–260	30–279
SLM *z*	−0.355	0.164	0.889	2.77	3.56
SLM *x–y*	−0.471	0.098	0.767	2.58	3.34
Invar 36^®^	1.6^a^	2.0	–	4.1	–
SLM stress-relieved *z*	1.00	1.34	1.85	2.96	3.68
SLM stress-relieved *x–y*	0.70	0.99	1.53	2.74	3.36

^a^For temperature range 25–93 °C

Units are (10^−6^/ °C); uncertainty of measurement is ±0.01 10^−6^/ °C

It is noted from Fig. 5 that the normal linear thermal expansion begins at approximately 240 °C for both orientations; this is taken as the temperature at which the magnetovolume phenomenon is fully overcome by atomic vibrations. However, this does not indicate a conflicting Curie point to that of the literature, and it is merely the temperature at which the phenomenon’s effect becomes negligible.

Temperature ranges for *α*_CTE_ measurements shown in Table 2 were chosen for the best comparison with the literature values. The final temperature range represents room to Curie temperature.

## Observed reduction of thermal expansion coefficient

In comparison with Invar36^®^, as-deposited SLM Invar displays significantly lower CTE values for all temperature ranges and negative CTE for 30–100 °C. The initial consideration for this variance was differences in chemical composition; however, the reported chemical compositions of LPW-Invar and Invar36^®^ are similar [2]. In addition, the CTE of an alloy is only sensitive to changes in chemical composition of the order of 5 wt% and greater [18]. Conversely, the ‘Invar effect’ is very sensitive to changes in concentration of ferromagnetic elements Fe, Ni and Co, but no quantitative relationships for this sensitivity exist. Given that the elemental constituents of the LPW-Invar do not vary from the standardised composition—i.e. additions of Co—and that any deviations from the 64Fe–36Ni form would reduce the Invar effect rather than boost it [1], this is again not considered to be a significant factor in the observed reduction of thermal expansion.

It was also considered that the unique microstructure of as-deposited SLM samples may have an effect on the thermal expansion. The thermal expansion of a crystal must possess the symmetry of the crystal [19], and therefore, cubic crystals, like the fcc of Invar, will exhibit isotropic thermal expansion in all crystallographic directions. It is therefore expected that, without external influence, a grain of Invar dendrites will expand homogenously and *α*_CTE_ will be the same regardless of its shape or size. If grain morphology did have an effect, dramatic variation between x-y and z measured *α*_CTE_ would be observed because of the high aspect ratio grains and columnar dendrites. Although a disparity has been observed, it is only marginal and therefore unlikely to be as a result of grain structure.

An investigation by Wang et al. [20] demonstrated that residual stress anisotropy can lead to effective CTE anisotropy. This could not only be the cause of the disparity but also potentially explain the observed negative, and or low, expansion of the SLM-fabricated samples.

To investigate this further, as-deposited SLM samples were stress-relieved by heat treatment. The samples were held at 850 °C for 1 h under an argon atmosphere to prevent oxidation. After the heat treatment, the thermal expansion analysis was conducted again for both x–y and z orientations. The stress relief resulted in an overall increase in thermal expansion coefficient for the samples shown in Table 2; however, the disparity between the test orientations increased in magnitude; see also Fig. 5 and Table 2. This implies that the residual stress was contributory to the exceptionally low CTE values in the as-deposited samples, but was not responsible for the anisotropy.

This behaviour is not in agreement with Qiu et al. who reported very similar *α*_CTE_ values for as-deposited and heat-treated material. However, Qiu employed a different heat treatment regime of multiple exposures with reducing temperature and increasing dwell time, and critically, the initial stress relieving cycle held the maximum temperature (830 °C) for half of the duration (0.5 h) compared to this work (1 h). The lower dwell time may have not been sufficient to fully stress-relieve the material, and therefore, the atomic movement was still being restricted.

**Figure 6:**
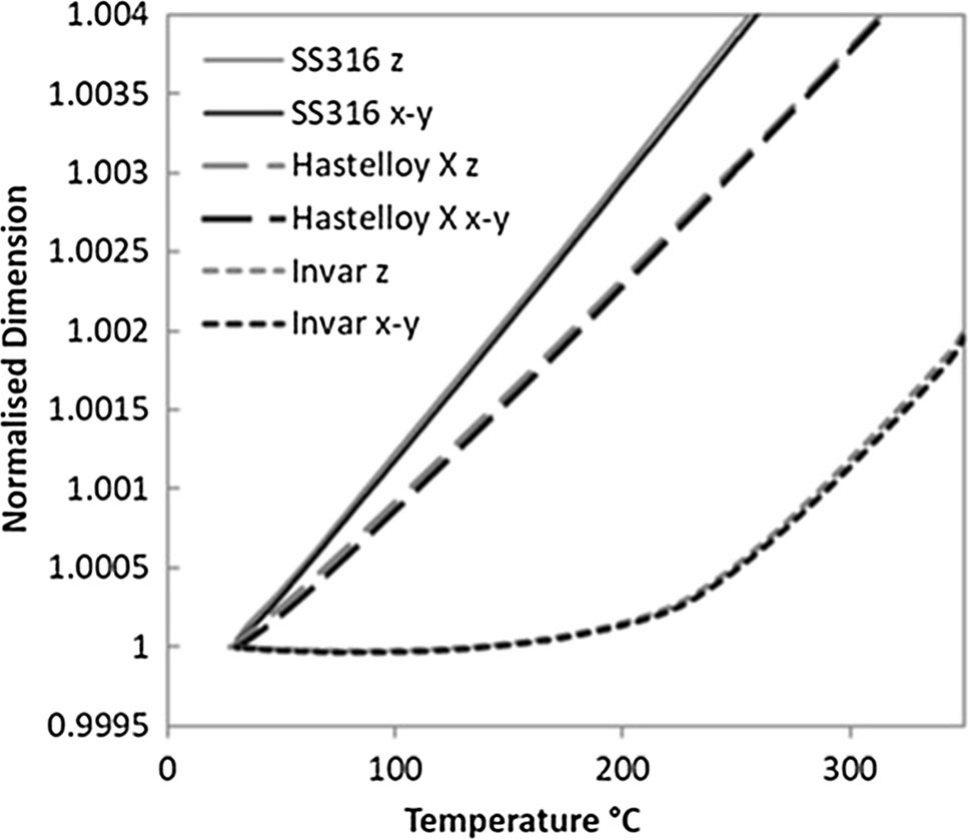
Normalised thermal expansion for x-y and z test orientations of SLM as-deposited stainless steel 316 and Hastelloy X, with Invar curves added for comparison.* Scaled view* shows marginal test orientation disparity across the sample range

To investigate whether reduction of thermal expansion occurred in other alloy systems when processed by SLM, as-deposited samples of stainless steel 316l (316l) and Hastelloy X were analysed. Thermal expansion analysis was performed for both x–y and z orientations, as with Invar; see Fig. 6. The values were then compared to those values for conventionally manufactured equivalents; see Table 3. Unlike Invar, the 316l and Hastelloy X samples did not display significantly different *α*_CTE_ than that reported for conventional manufacturing techniques. Although not quantified, it is expected that both alloys will be under significant residual stress in the as-deposited state [21] and likely that this residual stress will be greater than that for Invar, given their comparative yield strengths. The implication is therefore that although stress-relieved Invar does display a higher CTE than that of the as-deposited material, the residual stress does not affect the thermally driven expansion of the lattice but rather the magnetovolume contraction. Observations of the thermal expansion curves in Fig. 5 support this, where, beyond the range of the Invar effect’s influence (*T* > 280 °C), the as-deposited and stress-relieved Invar expand at the same rate .

**Table 3:** Mean coefficient of thermal expansion *α*_CTE_ for SLM-fabricated stainless steel 316l (316l), Hastelloy X (Hast X) and literature values of the same alloys fabricated from conventional techniques [22, 23]

Temp (°C)	30–100	30–200	30–300	30–400	30–500
SLM 316l *z*	17.27	17.48	17.76	17.93	18.17
SLM 316l *x–y*	16.63	17.21	17.51	17.64	17.57
Cold-rolled 316l	16	16.5	17	17	18
SLM Hast X *z*	12.87	13.57	13.98	14.28	14.55
SLM Hast X *x–y*	12.59	13.52	14.02	14.35	14.65
Sheet Hast X	13	13.5	13.8	14.5	14.5

Units are (10^−6^/ °C)

A consistent disparity between the test orientations was observed for 316l but not Hastelloy X. In Hastelloy X, the z orientation displays the larger *α*_CTE_ values up to 300 °C, after which point the x–y orientation measurements become greater. Most notable though is that the difference in *α*_CTE_ between orientations for all three alloys does not vary greatly from 0.5 × 10^−6^/ °C. The implication is therefore that the disparity is a process-related phenomenon, and is not specific to Invar.

The only other feature which varies specifically with orientation is defect population. Given the fine columnar grain structure and fabrication through layering of powder, it is plausible that defects such as micropores and microcracks will be aligned with either scan direction or grain orientation. Even if the expansion of the individual defect is isotropic, a preferential concentration of defects in a particular orientation will result in bulk anisotropy. This would also explain the small absolute magnitude of disparity and why it remains consistent with increasing temperature and not affected by the magnetovolume Invar effect.

## Conclusions and summary

Full-density (>99.5% dense) components of Invar were fabricated after optimisation of laser scan parameters. The optimised parameters were then used to build tensile and thermal expansion test components. SLM-fabricated Invar displayed tensile properties comparable to that cold-drawn, and superior to annealed, Invar36^®^.

As-deposited SLM-fabricated Invar was shown to have a lower CTE than conventionally manufactured Invar36^®^. In addition to this, a small but consistent anisotropy in the CTE was observed between the x–y and z test orientations for the as-deposited SLM Invar samples. After considering the effects of microstructure and residual stress, the anisotropy was attributed to heterogeneous distribution of defects within the as-deposited components.

Residual stress was found to be a significant contributor to the reduced CTE of SLM-fabricated Invar. However, comparisons of thermal expansion behaviour between other Fe–Ni alloy systems, and stress-relieved Invar samples, implied that the influence of process-induced residual stress was not universal. Rather, it affected the magnetovolume contraction (Invar effect) and not the thermally induced expansion of the lattice.

To summarise, SLM-fabricated Invar displays comparable tensile properties to those fabricated by conventional processes. The SLM process does not affect the low thermal expansion properties of Invar in a negative way; instead, it produces a further reduced thermal expansion coefficient for atmospheric temperatures.
